# Endoscopic transcecal resection of a low grade appendiceal mucinous neoplasm

**DOI:** 10.1055/a-2462-1559

**Published:** 2024-12-03

**Authors:** Xiwei Ding, Shanshan Shen, Lei Wang

**Affiliations:** 1 66506 Department of Gastroenterology, Nanjing Drum Tower Hospital, The Affiliated Hospital of Nanjing University Medical School, Nanjing, China

Appendiceal mucinous neoplasms are rare tumors for which clinical management is challenging. Endoscopic transcecal appendectomy has been shown to be effective for the treatment of appendiceal orifice lesions 1 2 3 . Herein, we report the application of this technique to successfully remove an appendiceal mucinous neoplasm without adverse events.

The patient, a 54-year-old woman, underwent colonoscopy because of a change in bowel habit. A submucosal tumor-like mass was found in the appendiceal orifice ( Fig. 1 ). Endoscopic ultrasonography performed at another hospital had revealed a low echogenic mass originating from the submucosal layer, so an appendiceal neoplasm was suspected.

**Fig. 1:**
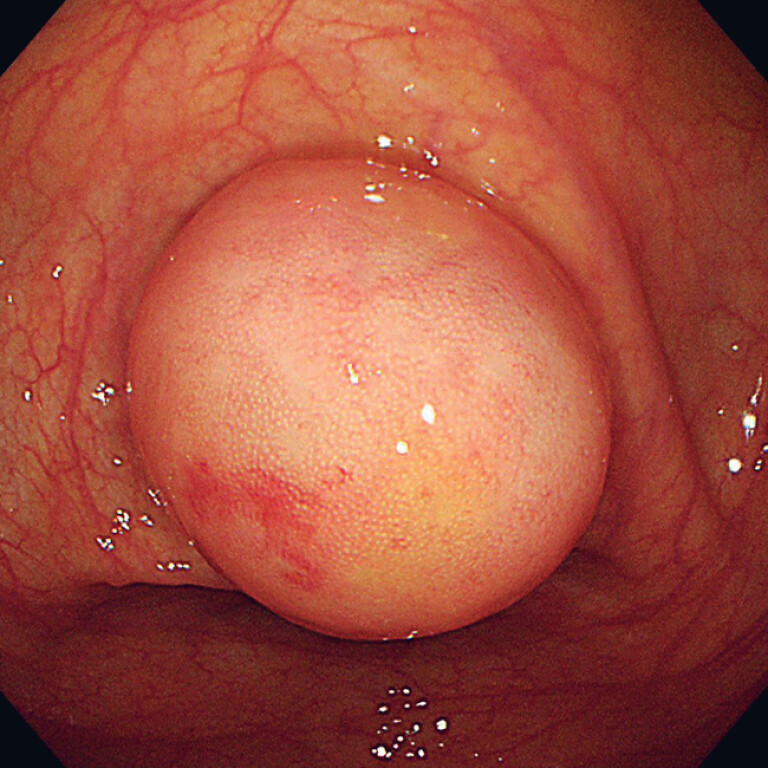
Endoscopic view showing a submucosal tumor-like lesion in the appendiceal orifice.

The patient refused to undergo surgery. Therefore, after informed consent had been obtained, endoscopic transcecal resection of the appendix together with the lesion was successfully performed by an experienced endoscopist ( Video 1 ). This involved the following steps: (i) a half-circumferential endoscopic full-thickness incision was made around the appendiceal lesion using a DualKnife and IT knife; (ii) the appendix was separated from the mesoappendix using the IT knife; (iii) circumferential full-thickness resection was performed around the lesion using the IT knife; (iv) after the lesion had been removed ( Fig. 2 ), the cecal defect was closed with endoscopic clips ( Fig. 3 ). The procedure time was 90 minutes. The patient received prophylactic antibiotic therapy, and was given a liquid diet, starting 72 hours after the procedure. She was discharged 5 days after the procedure and no adverse events occurred.

Endoscopic transcecal resection of an appendiceal mucinous neoplasm.Video 1

**Fig. 2:**
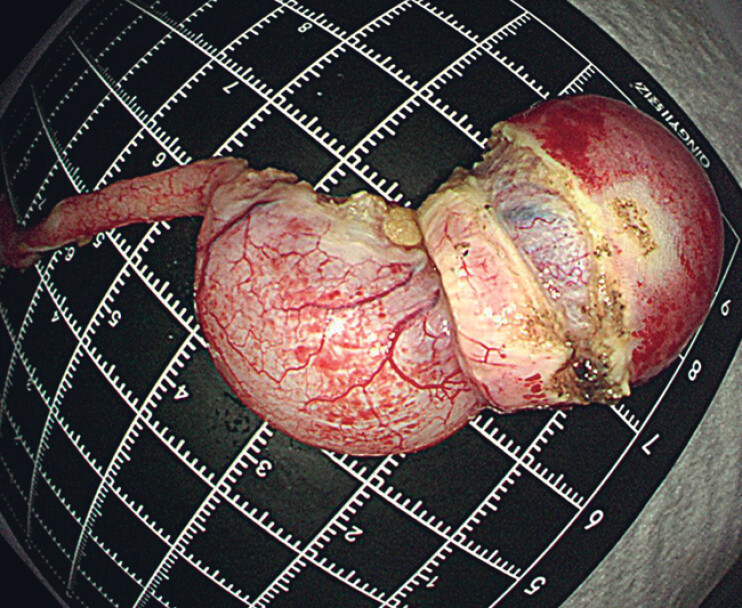
Macroscopic appearance of the intact specimen.

**Fig. 3:**
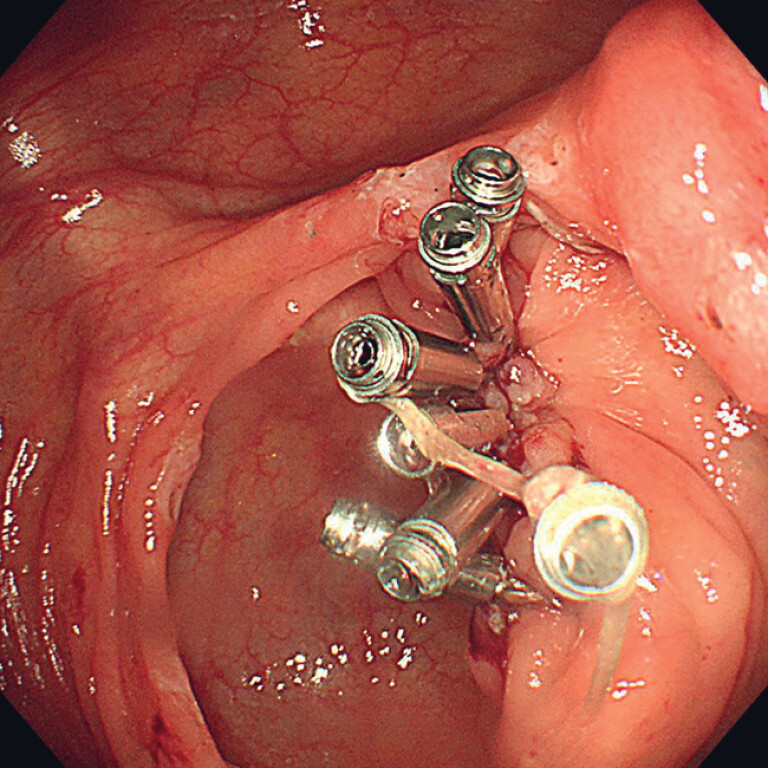
Endoscopic view of the cecal defect after its closure with endoscopic clips.

On dissection of the lesion in vitro, a large amount of mucinous substance was seen within the lesion ( Fig. 4 ). Pathology showed a low grade appendiceal mucinous neoplasm with negative margins ( Fig. 5 ). The tumor size was 3 × 2.5 × 2.3 cm.

**Fig. 4:**
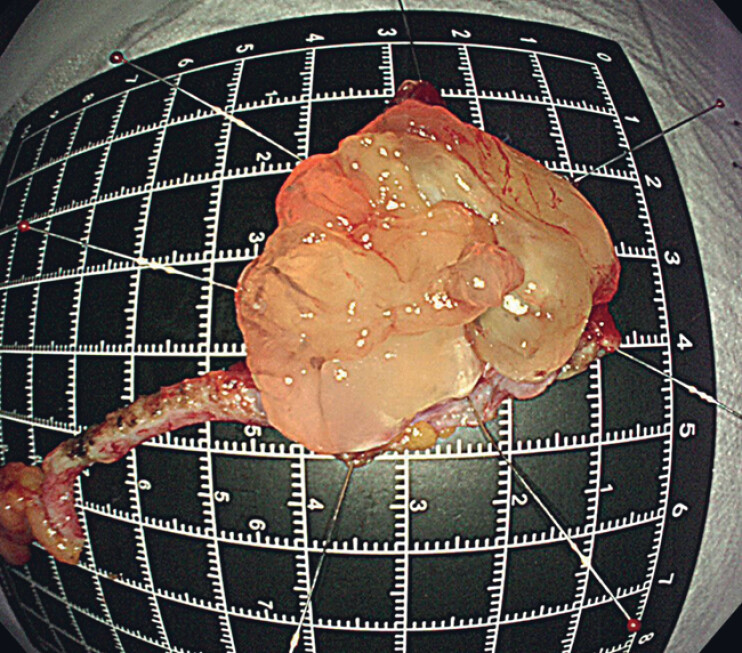
Macroscopic appearance of the specimen after incision.

**Fig. 5:**
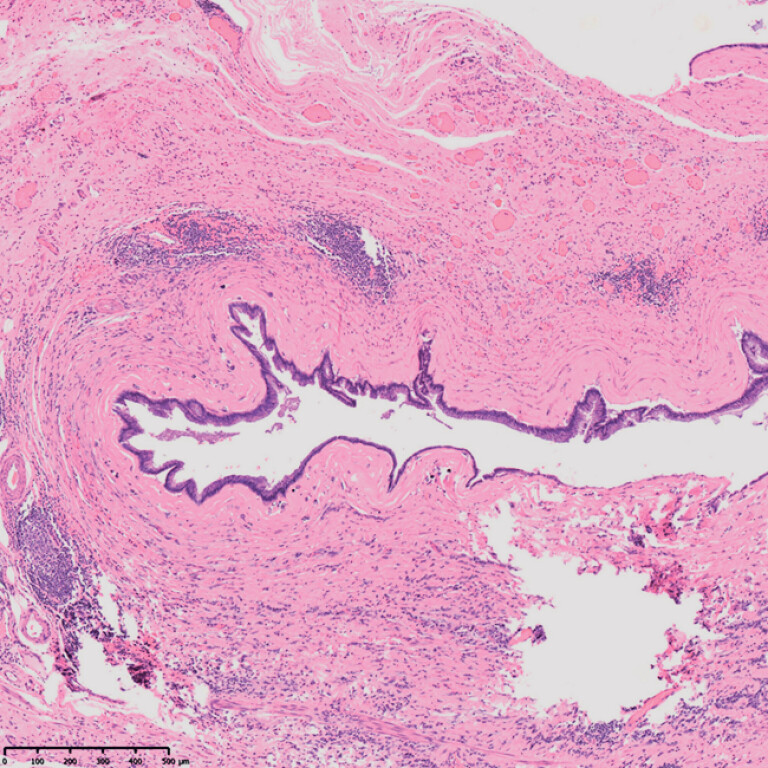
Histopathologic view showing a low grade appendiceal mucinous neoplasm.

Our case shows that endoscopic transcecal appendectomy may provide a new option for the treatment of appendiceal mucinous neoplasms confined to the appendiceal wall.

Endoscopy_UCTN_Code_TTT_1AQ_2AD_3AF

## Notes

Endoscopy E-Videos https://eref.thieme.de/e-videos
*E-Videos* is an open access online section of the journal *Endoscopy* , reporting on interesting cases and new techniques in gastroenterological endoscopy. All papers include a high-quality video and are published with a Creative Commons CC-BY license. Endoscopy E-Videos qualify for HINARI discounts and waivers and eligibility is automatically checked during the submission process. We grant 100% waivers to articles whose corresponding authors are based in Group A countries and 50% waivers to those who are based in Group B countries as classified by Research4Life (see: https://www.research4life.org/access/eligibility/ ). This section has its own submission website at https://mc.manuscriptcentral.com/e-videos .
